# Small steps towards an EU health information system

**DOI:** 10.1186/s13690-018-0280-6

**Published:** 2018-06-28

**Authors:** Hans van Oers, Herman van Oyen

**Affiliations:** 1 National Institute of Public Health and the Environment (RIVM), Bilthoven, The Netherlands; 2 Epidemiology and public health, Sciensano, Brussels, Belgium

A Health Information System (HIS) can be defined as the necessary infrastructure for the monitoring of health activities and population health outcomes. It includes the collection, analysis, interpretation and inference, storage, transmission, display, dissemination, and further utilization of data and information from various sources. It is a complex, multi-layered system, aimed at producing health intelligence at local, national and international level. An international HIS has two major purposes. First, it aims to inform the agenda of international organizations that run different HISs, to monitor progress towards agreed targets. Second, it supports cross-country learning through international comparisons and sharing of information about effective policy actions.

Until now the European Commission (EC) and European States have practically failed to set up such a HIS covering all EU Member States. There is no single comprehensive EU-wide HIS that allows monitoring of agreed targets or that supports cross-country comparisons effectively. The current EU HIS has developed through investments in individual and independent EU projects, and through the work of the EC and international organisations including the World Health Organisation and the Organisation for Economic Co-operation and Development. What was established over the last decades in the EU is a wide range of EU health information structures and services, with fragmented EU databases and registries, lacking cohesion and sustainability, and resulting in substantial health information inequalities between EU Member States. Current vertical and fragmented EU HIS hampers the production of relevant information to support public health policymakers at regional, national and EU-level. Furthermore, international comparisons to stimulate cross-country learning are complex, incomplete and time-consuming, and sometimes even impossible.

Recently, new steps are taken for the realization of an overarching EU HIS, by means of the EU-funded BRIDGE Health project. This project brought together good examples, experiences and practices of EU-wide collaboration in the field of health information. The overarching goal of BRIDGE Health was to explore the possibilities to create an organisational entity that could take up the tasks that come with the need for the further development of an EU HIS.

In this thematic series on Health Information, results of a number of studies that were performed within the framework of BRIDGE Health are presented. These studies show the wide diversity of subjects that can be placed under the flag of a health information system. Moreover, they also show the added value and relevance of cross-country collaboration in this field. The different manuscripts guide the reader through the most recent achievements and the lasted evolutions within the EU – small steps towards an EU HIS. BRIDGE Health ended in October 2017, but is followed up by a new Joint Action on Health Information (InfAct). InfAct focusses on involving Ministries of Health and Research in the further developments and concretizing of an EU HIS through an European Research Infrastructure Consortium on Health Information for Research and Evidence-based Policy (HIREP-ERIC). One of the specific outputs of InfAct is to have a political commitment of the EU Member States through a Memorandum of Understanding to invest in and start the development of an EU health information system.

